# First person – Michael Koch

**DOI:** 10.1242/dmm.052244

**Published:** 2025-01-02

## Abstract

First Person is a series of interviews with the first authors of a selection of papers published in Disease Models & Mechanisms, helping researchers promote themselves alongside their papers. Michael Koch is first author on ‘ Dose-dependent effects of Nrf2 on the epidermis in chronic skin inflammation’, published in DMM. Michael conducted the research described in this article while a PhD candidate and research scientist in Professor Sabine Werner's lab at ETH Zürich, Zürich, Switzerland. His research interests include determining how dysregulation of complex networks on a molecular level leads to disease on an organ level, and how we can target those dysregulations to develop novel therapeutic approaches.


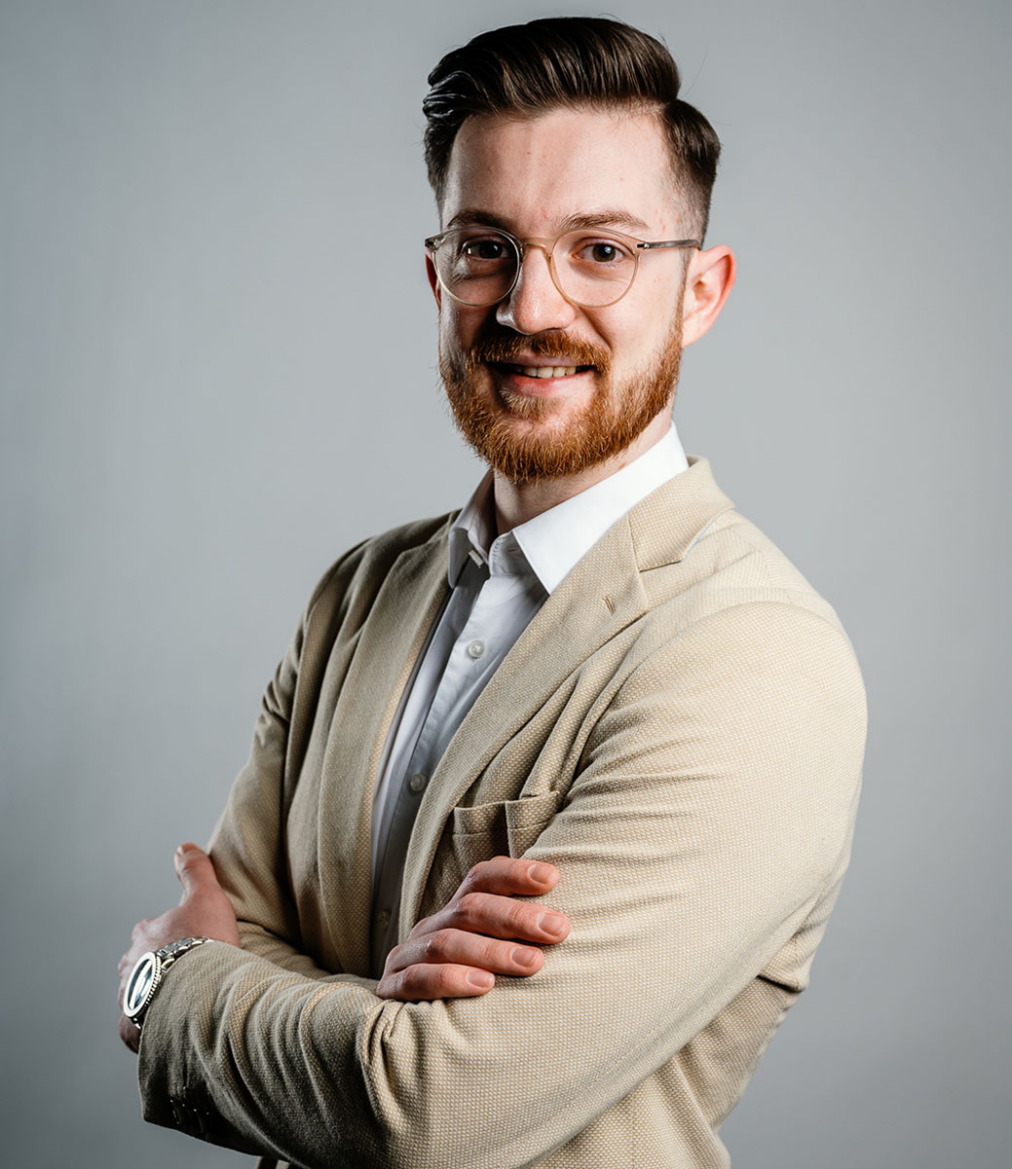


**Michael Koch**

**Who or what inspired you to become a scientist?**

Curiosity was the driving force behind my decision to become a scientist. Rather than accepting things as they are with a simple “that's just the way it works”, I am motivated to question how and why things function the way they do. In addition, I need a link to clinical relevance. The possibility to have a positive impact on future patients’ lives has become another major driver and feels greatly rewarding.

**What is the main question or challenge in disease biology you are addressing in this paper? How did you go about investigating your question or challenge?**

Atopic dermatitis (AD) is a common chronic inflammatory skin disease, characterized by an impaired epidermal barrier and immunological alterations. While we could previously show that AD patients exhibit decreased epidermal activity of the transcription factor NRF2, it was still unclear how this alteration contributes to the pathophysiology of the skin disease. To approach this question, we utilized mice lacking fibroblast growth factor receptors 1 and 2 in keratinocytes (K5-R1/R2 mice), because they recapitulate AD-like symptoms. Additional keratinocyte-specific loss or gain of function of Nrf2 in these mice allowed us to investigate the role and function of Nrf2 in an AD-like setting *in vivo*.

**How would you explain the main findings of your paper to non-scientific family and friends?**

The skin functions as a barrier that protects us from the environment. If this protective layer doesn't work properly, it can lead to a chronic skin disease called AD, which is associated with dry skin, intense itch and rashes. Here, we looked at a natural protector of the skin, called NRF2. In healthy skin, NRF2 helps keep the skin strong and repairs damage. In AD, NRF2 doesn't work as well, which may make the skin weaker. Using mice with skin problems similar to AD, we found that when Nrf2 wasn't active enough, the skin showed more damage. Boosting Nrf2 for a short time helped protect the skin. However, turning it on too much or for too long caused additional problems, like overly thickened skin, increased damage and a disturbed immune response. Nrf2 therefore acts like a thermostat for skin health – too little activity makes the skin vulnerable, but turning it up too high can create new problems. To maintain skin health, the activity of Nrf2 must be carefully balanced.

[…] limited activation of NRF2 in the skin of AD patients might serve as a potential therapeutic approach to protect the skin and improve barrier function.

**What are the potential implications of these results for disease biology and the possible impact on patients?**

Although several new and efficient AD therapeutics have recently entered the market, they mainly aim at symptom management, calling for the need to develop alternative treatments. Here, we give an insight into the complex role of NRF2 in AD. We show that decreased Nrf2 activity contributes to DNA damage and senescence in the epidermis of an AD-like mouse model. Through limited pharmacological activation, we can partially rescue the barrier defect, a hallmark of AD skin. Our findings suggest that limited activation of NRF2 in the skin of AD patients might serve as a potential therapeutic approach to protect the skin and improve barrier function. Simultaneously, we show that extended NRF2 activation can have detrimental effects and should be avoided in AD patients. Interestingly, NRF2-activating fumaric acid esters are already in use for the treatment of psoriasis, another inflammatory skin disease.

**Figure:**
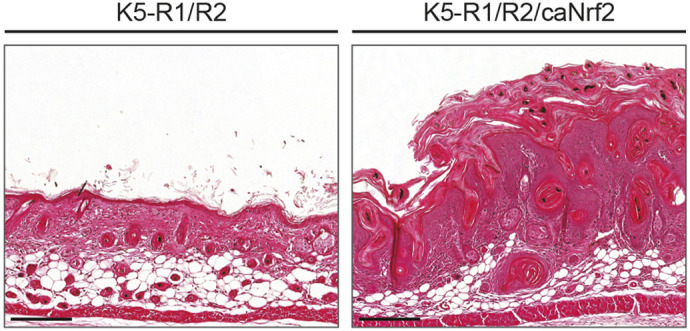
**Hematoxylin and Eosin staining on back skin sections of K5-R1/R2 and K5-R1/R2/caNrf2 mice, showing hyperkeratosis and severely thickened viable epidermis and stratum corneum in the latter.** Scale bars: 100 μm.

**Why did you choose DMM for your paper?**

DMM is a highly regarded biomedical journal. We believe that the questions we approached in our research paper greatly align with the focus of the journal. In addition, I had been co-author of another article previously published in DMM and the publication process was fair, transparent and, overall, very positive. We value the possibility to publish in DMM.

**Given your current role, what challenges do you face and what changes could improve the professional lives of other scientists in this role?**

When engaging with non-scientists, I frequently experience a lack of understanding and/or appreciation for the work we do as scientists. To address this, we need to actively promote open science, not only to share our findings more widely but also to highlight the critical role of basic research in driving innovation and solving real-world problems. Efforts like open-access publishing and initiatives to communicate science in relatable ways are important steps toward bridging this gap, but meaningful progress will require sustained effort over time. By making research more accessible and transparent, we can foster greater public appreciation and engagement while also enhancing collaboration and accountability within the scientific community.

By making research more accessible and transparent, we can foster greater public appreciation and engagement while also enhancing collaboration and accountability within the scientific community.

**What's next for you?**

I want to utilize the skills and knowledge that I gained during my PhD to move one step closer to the patient. Therefore, I want to contribute to pushing the next generation of therapeutics of challenging diseases by being involved in drug discovery and pre-clinical development.

**Tell us something interesting about yourself that wouldn't be on your CV**

When I'm not in the lab, I enjoy building 3D-printed and Arduino-based projects. Also, I like to get active by boxing or hiking.
